# The Role of Sfp1 in *Candida albicans* Cell Wall Maintenance

**DOI:** 10.3390/jof8111196

**Published:** 2022-11-13

**Authors:** Che-Kang Chang, Min-Chi Yang, Hsueh-Fen Chen, Yi-Ling Liao, Chung-Yu Lan

**Affiliations:** 1 Institute of Molecular and Cellular Biology, National Tsing Hua University, Hsinchu 30013, Taiwan; 2 Department of Life Science, National Tsing Hua University, Hsinchu 30013, Taiwan; 3 School of Medicine, National Tsing Hua University, Hsinchu 30013, Taiwan

**Keywords:** *Candida albicans*, cell wall integrity, cell wall stress response, Sfp1, Cas5

## Abstract

The cell wall is the first interface for *Candida albicans* interaction with the surrounding environment and the host cells. Therefore, maintenance of cell wall integrity (CWI) is crucial for *C. albicans* survival and host-pathogen interaction. In response to environmental stresses, *C. albicans* undergoes cell wall remodeling controlled by multiple signaling pathways and transcription regulators. Here, we explored the role of the transcription factor Sfp1 in CWI. A deletion of the *SFP1* gene not only caused changes in cell wall properties, cell wall composition and structure but also modulated expression of cell wall biosynthesis and remodeling genes. In addition, Cas5 is a known transcription regulator for *C. albicans* CWI and cell wall stress response. Interestingly, our results indicated that Sfp1 negatively controls the *CAS5* gene expression by binding to its promoter element. Together, this study provides new insights into the regulation of *C. albicans* CWI and stress response.

## 1. Introduction

*Candida albicans* is part of the normal microbiota of humans, commonly inhabiting the skin and mucosal surfaces of healthy persons. However, *C. albicans* is also an opportunistic pathogen and can cause superficial to life-threatening systemic infections, particularly in immunocompromised patients. Epidemic studies indicated that *C. albicans* along with other *Candida* species is one of the leading causes of hospital-acquired bloodstream infections (aka candidemia), and the mortality of candidemia can be as high as 46–75% [1,2]. Moreover, the emergence of antifungal drug resistance in *Candida* also is of great concern in clinical settings [3].

The cell wall is the outermost structure of *C. albicans* and is vital to provide protection against environmental stresses and interact with the host cells. The *C. albicans* cell wall is a two-layer structure composed of ~10% glycoproteins and ~90% polysaccharides, which include mannan, glucan, and chitin [4,5,6]. The inner layer is constituted mainly by β-1,3-glucans, β-1,6-glucans and chitin [7]. Nevertheless, the outer layer of the cell wall is enriched with mannan, which is incorporated into mannoprotein and phosphomannan [7]. Importantly, the composition and structure of the cell wall are highly correlated with pathogenicity of *C. albicans*. For example, cell wall proteins such as Als1, Als3, and Hwp1 mediate *C. albicans* adhesion and invasion of host cells and tissues [8,9,10,11]. In addition, the outer mannan layer masks the antigenic β-1,3-glucans to diminish their recognition by host immune cells [12]. Finally, the cell wall is an important therapeutic target for antifungal drugs such as echinocandins [13]. Together, the cell wall is essential for *C. albicans*, and the integrity of the cell wall requires to be monitored constantly.

Although the cell wall is a complex structure, its organization is dynamic. In response to environmental stresses, *C. albicans* remodels its cell wall to prevent cell damage and to adapt to environmental changes. Cell wall remodeling is a process that involves different signaling pathways and transcription factors. For example, glucose and lactate mediate osmotic tolerances and cell wall remodeling in *C. albicans*, and the calcineurin signaling pathway regulates this carbon source-induced remodeling [14]. As another example, the echinocandins (e.g., caspofungin) target *C. albicans* cell wall by inhibiting β-1,3-glucan synthesis [14]. *C. albicans* cells exposed to caspofungin also undergo cell wall remodeling [15]. Intriguingly, several transcription factors are involved in the control of caspofungin-induced cell wall stress response in *C. albicans* [16]. Among these regulators, *C. albicans* Cas5 is a unique zinc finger transcription factor, which lacks an ortholog in *Saccharomyces cerevisiae* and in most other eukaryotes [16,17]. In addition, Cas5 not only governs the expression of many cell wall integrity (CWI) and caspofungin-responsive genes but also modulates cell cycle dynamics in *C. albicans* [16,17,18]. Lastly, previous studies demonstrated that the human antimicrobial peptide LL-37 also targets the cell wall of *C*. *albicans* and causes cell wall remodeling [19,20,21,22]. Recently, we found that unfolded protein response (UPR) signaling related to the endoplasmic reticulum is activated in this LL-37-induced cell wall stress response [23].

Sfp1 is a C2H2-type zinc finger transcription factor with multiple functions in *C*. *albicans*. Sfp1 regulates ribosomal gene expression and carbon-conditional stress adaptation [24,25]. Moreover, the *SFP1*-deletion (*sfp1*∆/∆) mutant exhibits increased *C*. *albicans* cell adhesion and biofilm formation and is more resistant to oxidants, macrophage-mediated killing, and reactive oxygen species (ROS)-generating antifungals [24,26]. Interestingly, Sfp1 also plays a role in the LL-37-induced cell wall stress response [23].

Because Sfp1 contributes to cell wall stress responses, it raises the possibility that Sfp1 may have an impact on CWI. In this work, we thus correlated the function of Sfp1 and CWI maintenance, and also explored the relationship between Sfp1 and Cas5. The results showed that deletion of *SFP1* affected cell susceptibility to cell wall-disrupting agents and cell surface hydrophobicity (CSH). The *sfp1*∆/∆ mutant was resistant to caspofungin. Moreover, the *sfp1*∆/∆ mutant also exhibited altered cell wall composition and structure, and modulated expression of cell wall biosynthesis and remodeling genes. Finally, we further showed that Sfp1 interacts with the *CAS5* gene promoter and regulates its gene expression. Our findings reveal a previously unknown function of Sfp1 in regulation of *C. albicans* CWI.

## 2. Materials and Methods

### 2.1. C. albicans Strains and Growth Conditions

*C. albicans* strains used in this study are listed in Table S1 (in the Supplementary Materials). Cells were grown in YPD medium (1% yeast extract, 2% peptone and 2% glucose) and synthetic complete (SC) medium (0.67% yeast nitrogen base [YNB] with ammonium sulfate, 0.079% complete supplement mixture, and 2% glucose). Plates were prepared with 1.5% Bacto-agar. All reagents used were purchased from Sigma-Aldrich (St. Louis, MO, USA) unless indicated otherwise. One colony of *C. albicans* was inoculated into YPD broth and grown overnight at 30 °C with shaking (180 rpm). The overnight culture was harvested by centrifugation and washed with sterile double-distilled water (ddH_2_O). Cells were then subcultured in SC medium (with an initial optical density at 600 nm [OD_600_] of 0.5) and grown to the exponential phase for further experiments. A cell number of ~1 × 10^7^ cells per 1 OD_600_ unit was determined by cell counting.

### 2.2. Strain Construction in C. albicans

Construction of gene deletion and reintegration strains was performed using the *SAT1*-flipper method [27]. The primers used are listed in Table S2 (in the Supplementary Materials). To construct the *CAS5*-deletion (*cas5*Δ/Δ) strain, the 5′ flanking region of *CAS5* was amplified from the SC5314 genome using the primer pair CAS5UR-F-KpnI and CAS5UR-R-XhoI. In addition, the 3′ flanking region of *CAS5* was amplified from the SC5314 genome using the primer pair CAS5DR-F-SacII and CAS5DR-F-SacI. The resulting 5′ and 3′ flanking regions of *CAS5* were independently cloned into the plasmid pSFS2A [27]. The DNA fragment carrying these flanking regions of *CAS5* and the *SAT1*-flipper cassette was excised via *Kpn*I/*Sac*I digestion, followed by purification. This linear DNA was then transformed into *C. albicans* cells for integration into the chromosome. The transformants were selected for nourseothricin resistance and verified by PCR [24]. To pop out the *SAT1*-flipper cassette from the *CAS5* locus, the cells were grown in YPM medium (1% yeast extract, 2% peptone, and 2% maltose) to induce the *MAL2* promoter-regulated recombinase for *SAT1*-FLIP excision. The heterozygous *CAS5*-deletion mutants (*cas5*Δ/*CAS5*) were used for the second round of deletion cassette integration and excision to knock out the second allele of *CAS5* (*cas5*Δ/Δ). Similar procedures were used to delete *CAS5* in the homozygous *SFP1*-deletion background, generating the *sfp1*Δ/Δ *cas5*Δ/Δ double deletion mutant.

To generate *C*. *albicans* strain that expressed HA-tagged Sfp1, the LOB301 vector was used as previously described [28]. Briefly, the *SFP1* gene was PCR-amplified from the SC5314 using the primer pair SphI-HA-SFP1-F and SphI-HA-SFP1-R (Table S2). The PCR product was then cloned into the LOB301 vector to generate LOB301-HA-SFP1. The DNA fragment carrying the 5′ and 3′ flanking regions of *ENO1*, the *SAT1*-flipper cassette, and HA-*SFP1* were excised from LOB301-HA-SFP1 via *Sac*I digestion. The linear DNA was purified and transformed into the *sfp1*Δ/Δ cells for integration into the chromosome between the 5′ and 3′ flanking sequences of *ENO1* via homologous recombination. Finally, the cells were grown in YPM medium to pop out the *SAT1*-flipper cassette.

### 2.3. Susceptibility to Cell Wall-Disrupting Agents

Cell susceptibility to cell wall-disrupting agents was examined. For the spot assay, *C. albicans* cells were grown overnight and harvested by centrifugation, washed with sterile ddH_2_O, and diluted to OD_600_ = 1. Cells were 10-fold serially diluted and 5 µL of each sample was spotted onto SC agar plates with or without congo red (100 μg/mL) and calcofluor white (600 μg/mL). The plates were incubated at 30 °C for 5 d and photographed. One representative image of three independent experiments with similar results (Figure S1 in the Supplementary Materials) is shown in the figure.

### 2.4. Zymolyase Sensitivity Assay and Assessment of Cell Surface Hydrophobicity (CSH)

Sensitivity to zymolyase was assayed as previously described [29]. Briefly, early-exponential-phase cells were harvested, diluted to OD_600_ = 2, and treated with 10 mM Tris-HCl (pH 7.5) containing 2 μg/mL Zymolyase 100T (Fisher Scientific, Waltham, MA, USA). To delineate the effect of zymolyase on cell lysis, the OD_600_ was measured every 30 min for 1.5 h.

CSH was determined as previously described [30] with some modifications. Cells were grown overnight, harvested, resuspended in 3 mL of phosphate-buffered saline (PBS), and diluted to OD_600_ = 1 (A_0_). Xylene (200 μL) was added to each 3 mL cell suspension and mixed well. The samples were incubated at 30 °C for 10 min, vortexed for 1 min, and kept at room temperature for 20 min to separate xylene from the aqueous phase. The aqueous phase was carefully collected and its OD_600_ was measured (A_1_). The percentage of CSH was calculated with the following formula: CSH (%) = [1 − (A_1_/A_0_)] × 100 [30].

### 2.5. Cell Response to the Antifungal Drug Caspofungin

To determine cell response to caspofungin, spot assay and measurement of minimum inhibitory concentrations (MICs) were performed. The spot assay was conducted as described above. For measuring MICs, the EUCAST broth microdilution method was used [31] with some modifications. Briefly, cells were grown in RPMI 1640 medium buffered with morpholinepropanesulfonic acid (MOPS) to pH 7.0 and supplemented with 1.8% glucose and a range of concentrations of caspofungin (0.0079 to 8 μg/mL). The OD_600_ was then measured after 24 h of incubation. The MIC50 and MIC90 were determined as the lowest concentration of caspofungin at which 50% and 90% of cells were inhibited compared with the drug-free control [32]. All assays were repeated at least three times with three biological replicates.

### 2.6. Transmission Electron Microscopy (TEM)

Cells were fixed with 2.5% glutaraldehyde for 24 h, washed twice with 0.1 M PBS (pH 7.0), post-fixed with 2% osmium tetraoxide for 2 h in a series of graded ethanol solutions, and embedded in Spurr’s resin at 72 °C for 12 h. Ultrathin sections were prepared with a Leica UC7 ultramicrotome and the images were obtained using a Hitachi HT-7700 transmission electron microscope with an accelerating voltage of 100 kV.

### 2.7. Measurement of Cell Wall Polysaccharide Content

To measure the total content of cell wall polysaccharides, an acid hydrolysis method was performed as previously described [33] with some modifications. Briefly, cells were resuspended in TE buffer (10 mM Tris-HCl, 1mM EDTA-2Na, pH 8), containing acid-washed glass beads. Cells were then broken by vortexing for 30 s and kept on ice for 30 s. This process was repeated 8 times. The pellets of cell wall were collected by centrifugation and resuspended in 200 μL of TE buffer, followed by adding 1 mL of 72% (*v*/*v*) sulfuric acid and 200 μL of 5% (*w*/*v*) phenol solution. The mixture was incubated at room temperature for 10 min, followed by incubation overnight at 37 °C to fully hydrolyzed polysaccharides into monosaccharides. Two hundred microliters of each sample were placed in each well of a 96-well microplate, and absorbance at 490 nm (OD_490_) was determined based on standard glucose curve. For quantification of the content of mannan, glucan and chitin, each sample was analyzed as previously described [33,34], using a high-performance anion-exchange chromatography with pulsed amperometric detection (HPAEC-PAD; Dionex ICS-5000 system, Thermo Fisher Scientific).

### 2.8. RNA Extraction and Reverse Transcription Real-Time Quantitative PCR (qPCR)

Total RNA extraction and reverse transcription for cDNA synthesis were carried out as previously described [35]. Real-time qPCR was performed using the StepOne Plus^TM^ Real Time PCR System (Applied Biosystems, Waltham, MA, USA). The primers used are listed in Table S2. Briefly, each 15-µL reaction mixture contained 30 ng cDNA, 7.5 μL Power SYBR green master mixture (Applied Biosystems), and 20 mM of each forward and reverse primer. The reaction was performed with one cycle at 95 °C for 10 min, followed by 40 repeated cycles at 95 °C for 15 s and 60 °C for 1 min. The *PMA1* transcripts were used as an internal control for the qPCR. For each sample, the average CT (cycle threshold) values were determined from two independent experiments with three biological replicates. The relative fold change in the expression of each gene was calculated using the 2^−ΔΔCT^ method [36].

### 2.9. Chromatin Immunoprecipitation (ChIP)

The ChIP assay was conducted as previously described [28] with some modifications. *C. albicans* cells were cross-linked with 1% formaldehyde at room temperature for 10 min, followed by adding glycine to a final concentration of 250 mM to terminate the cross-linking reaction. Cells were washed and resuspended in the RIPA buffer (10 mM Tris-HCl [pH 7.5], 150 mM NaCl, 0.5 mM EDTA [pH 8.0], 0.05% sodium dodecyl sulfate [SDS], 1% sodium deoxycholate, 1% NP-40) supplemented with protease inhibitors. To lyse the cells, each sample was mixed with glass beads and vortexed for 30 s and placed on ice for 30 s; repeated 10 times. The chromatin was then sheared to an average size of less than 500 bp by a Bioruptor sonicator (Diagenode, Liege, Belgium), and 200 μL of the sheared chromatin was incubated overnight with 1 μg anti-HA tag antibody (GTX115044; GeneTex, Hsinchu, Taiwan) at 4 °C with rotation. The mixture was subsequently incubated with 20 μL of PureProteome^TM^ Protein G magnetic beads (Millipore, Burlington, MA, USA) at room temperature for 4 h with rotation. The magnetic beads were washed three times with low-salt wash buffer (20 mM Tris-HCl, 150 mM NaCl, 0.05% SDS, 1% Triton X-100, 2 mM EDTA), high-salt wash buffer (20 mM Tris-HCl, 500 mM NaCl, 0.05% SDS, 1% Triton X-100, 2 mM EDTA), and LiCl wash buffer (0.25 M LiCl, 1% NP-40, 1% sodium deoxycholate, 1 mM EDTA, 10 mM Tris-HCl). Then, the elution buffer (100 mM NaHCO_3_, 1% SDS) was used to elute DNA, and crosslinking was reversed by incubating the DNA samples with proteinase K at 65 °C overnight. Finally, samples were treated with phenol/chloroform/isoamyl alcohol, and DNA was precipitated with absolute ethanol.

DNA was quantified with qPCR using primers specific to the promoters of *CAS5* and *ADE2* (Table S1) as previously described [28] with some modifications. To normalize the PCR efficiency for different primers, the amount of promoter DNA amplified from 25 ng of input DNA was defined as one and those from the ChIP experiment were normalized accordingly [28]. Because Sfp1 should not interact with the *ADE2* promoter in vivo, the value of the *ADE2* DNA fragments in each immunoprecipitation was considered as the non-specific background and was used for normalization to determine the ratio of change for particular promoters from the same strain [28].

### 2.10. Statistical Analysis

The Shapiro-Wilk test was used to asses for normality using R4.2.1 in RStudio 2022.07.1 + 554 “Spotted Wakerobin” for Windows. The two-tailed Student’s t-test or the non-parametric Mann-Whitney U test was applied to determine significant differences between samples. Statistical significance was indicated with a *p*-value  < 0.05. The t-test and the Mann-Whitney U test were conducted in Microsoft Excel 2016 and the resulting graph was generated.

## 3. Results

### 3.1. The Cell Wall Properties Are Altered in the SFP1-Deletion Mutants

**Figure 1:**
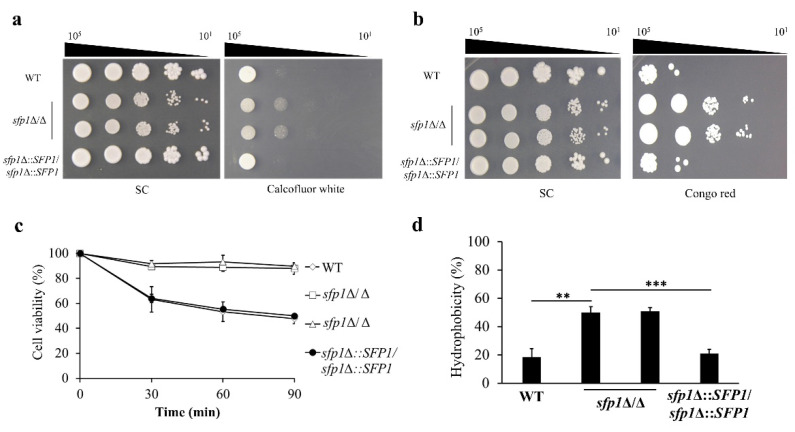
Deletion of *SFP1* leads to changes of *C. albicans* cell wall-related properties. The susceptibility of *C. albicans* to cell wall-disrupting agents calcofluor white (**a**) and congo red (**b**). The cells were ten-fold serially diluted and spotted onto SC agar plated with or without calcofluor white (600 μg/mL) and congo red (100 μg/mL). The plates were incubated at 30 °C for 5 d. Representative images of three independent experiments with similar results are shown. WT: wild type; (**c**) The zymolyase sensitivity assay. Cells were treated with 2 μg/mL of Zymolyase 100 T. Results are displayed as the mean ± standard deviation (SD) of two independent experiments; (**d**) Measurement of CSH. The CSH is expressed as percentage and displayed as the mean ± SD of three independent experiments. **, *p* < 0.01, ***, *p* < 0.001.

To test the possible connection between Sfp1 and CWI, cell wall properties were analyzed in the wild type (WT), *sfp1*Δ/Δ, and *SFP1*-reintegration strains. For the spot assay, cells were spotted onto agar plates with or without cell wall-disrupting agents congo red and calcofluor white. Congo red and calcofluor white bind to glucan and chitin respectively, and interfere with the synthesis and crosslinking of these polysaccharides [37]. As shown in Figure 1a,b, the *sfp1*Δ/Δ mutants were more resistant to congo red and calcofluor white than the WT and *SFP1*-reintegration strains. Moreover, cell sensitivity to zymolyase was also assessed. Zymolyase 100T is an enzyme mixture containing β-1,3-glucanase that degrades yeast cell wall polysaccharides, and zymolyase sensitivity reflects subtle differences in the cell wall β-glucan content. The results indicated that the *sfp1*Δ/Δ mutants were more resistant to zymolyase digestion (Figure 1c). Finally, CSH is associated with cell wall composition and architecture, cell adhesion, and biofilm formation in *C. albicans* [38,39]. Because the *SFP1* gene deletion affects *C. albicans* adhesion and biofilm formation [24], CSH of the WT, *sfp1*Δ/Δ and *SFP1*-reintegration strains were thus compared. In Figure 1d, a significantly higher percentage of CSH was obtained in the *sfp1*Δ/Δ mutants than in the WT and the *SFP1*-reintegration strains.

### 3.2. The SFP1-Deletion Mutant Has Increased Resistance to Caspofungin

**Figure 2:**
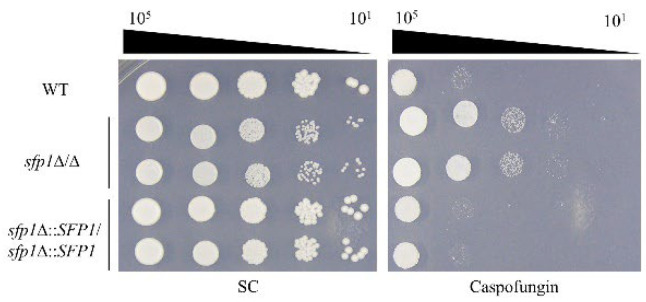
Deletion of *SFP1* confers resistance to the antifungal caspofungin. Caspofungin susceptibility testing by spot assay. The ten-fold serially diluted cells were spotted onto SC agar plates with or without caspofungin (8 μg/mL). The plates were incubated at 30 °C for 5 days. Representative images of three independent experiments with similar results are shown.

Caspofungin is an echinocandin antifungal drug, that targets fungal cell wall with potent activity against *Candida* infections [13,40]. To further determine the association of Sfp1 with CWI and cell wall stress response, cell susceptibility to caspofungin was also examined. For MIC determination, the *sfp1*Δ/Δ exhibited higher MIC50 and MIC90 for caspofungin than the WT and *SFP1*-reintegration strains (Table 1). In addition, deletion of *SFP1* rendered increased resistance to caspofungin as observed from the spot assay (Figure 2). Together, these results along with the findings that cell wall properties were altered in the *sfp1*Δ/Δ mutant suggest a role of Sfp1 in maintenance of *C. albicans* CWI. 

### 3.3. SFP1 Deletion Changes the Structure and Composition of the Cell Wall

**Figure 3:**
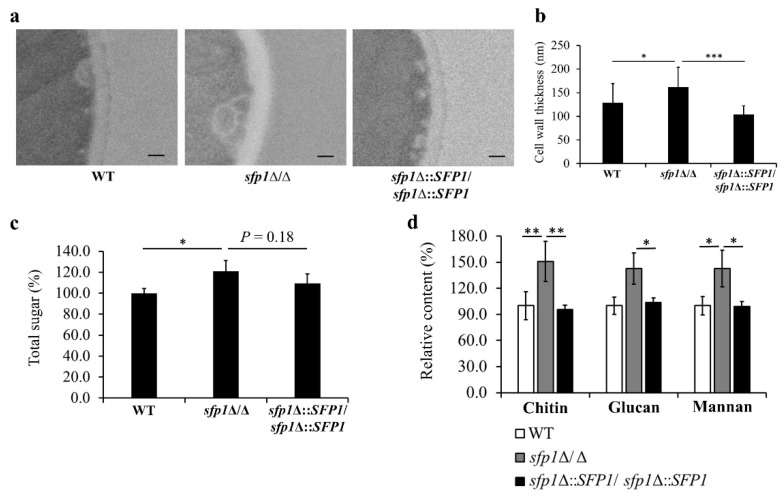
*SFP1* deletion also changes the cell wall and cell wall composition in *C. albicans*. (**a**) Cell wall thickness was examined by TEM and compared between the WT, *sfp1*∆/∆ and *SFP1*-reintegration strains. Representative images are shown. Scale bar: 100 nm; (**b**) For each *C. albicans* cells, 3 parts of cell wall were analyzed. Results are the mean ± SD from 20 individual cells. The statistical significance is assessed by Mann-Whitney U test. *, *p* < 0.05, ***, *p* < 0.001; (**c**) Total cell wall polysaccharide content was quantified. The wild type value was set as 100%. Results are displayed as the mean ± SD from three independent experiments. *, *p* < 0.05; (**d**) The cell wall chitin, glucan, and mannan content was quantified using HPAEC-PAD. Results are displayed as the mean ± SD of three independent experiments. *, *p* < 0.05, **, *p* < 0.01.

To further characterize the impact of *SFP1* deletion on the cell wall, cells were examined by TEM and cell wall thickness was determined. Representative images and quantitative data were displayed in Figure 3a,b, separately. The results showed that cell wall thickness was increased by ~25% in the *sfp1*Δ/Δ mutant compared to the WT and *SFP1*-reintegration strains. To better understand the changes in cell wall caused by *SFP1* deletion, cell wall polysaccharides were also quantified [33]. In Figure 3c, the total sugar content of cell wall was increased in the *sfp1*Δ/Δ mutant compared to the control strains. Moreover, the cell wall mannan, glucan, and chitin content was further assessed by HPAEC-PAD analysis, and was all largely increased in the *sfp1*Δ/Δ mutant compared to the control strains (Figure 3d). Interestingly, the increased cell wall content of polysaccharides is somehow consistent with the increased thickness of cell wall caused by the *SFP1* gene deletion. 

### 3.4. Sfp1 Controls the Cell Wall Biosynthesis and Remodeling-Related Genes

The cell wall integrity is maintained by remodeling through breaking and reforming chemical bonds between and within cell wall polysaccharides [41]. Different sugar synthases, hydrolases, and modification enzymes are involved in the cell wall remodeling process [14,42,43]. To determine whether Sfp1 can regulate genes related to cell wall biosynthesis and remodeling, expression of representative genes was detected using real-time qPCR. 

**Figure 4:**
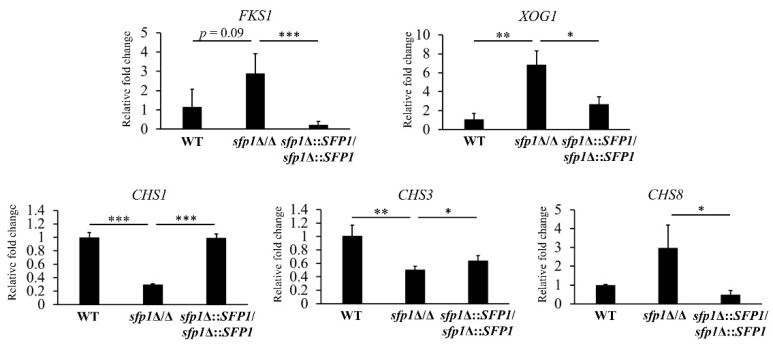
Sfp1 regulates expression of cell wall biosynthesis and remodeling genes. Gene expression was measured by real-time qPCR. The *PMA1* transcripts were used as an internal control for the RNA input. Results are displayed as the mean ± SD of three independent experiments. For each gene, the relative fold changes were displayed as the expression levels of an individual strain normalized to the WT strain (as 1). *, *p* < 0.05, **, *p* < 0.01, ***, *p* < 0.001.

As shown in Figure 4, expression of the *FKS1*, *XOG1*, *CHS1*, *CHS3,* and *CHS8* genes was greatly modulated in the *sfp1*Δ/Δ mutant compared to the WT and *SFP1*-reintegration strains. The *FSK1* gene encodes the catalytic subunit of β-1,3-glucan synthase which is a target of caspofungin [44,45]. For the *XOG1* gene, it encodes the major β-1,3-exo-glucanase that involves in cell wall remodeling, is a target of LL-37, and impacts cell susceptibility to inhibitors of chitin and glucan synthesis [14,21,46]. Importantly, Xog1 can shave the exposed β-1,3-glucan induced by different stresses, to facilitate immune evasion of *C. albicans* by reducing β-1,3-glucan accessibility to immune recognition [47]. Finally, the *CHS1*, *CHS3,* and *CHS8* genes encode a class II, class IV, and class I chitin synthase, respectively [48]. Chs1 is required for the synthesis of septum and for cell integrity [49,50]. Moreover, Chs3 synthesizes the short-chitin rodlets in the cell wall and Chs8 is responsible for synthesis of the long-chitin microfibrils in the septa [51]. Interestingly, *CHS* gene expression is also activated in cells treated with caspofungin and elevated chitin content affects *Candida* susceptibility to caspofungin [52,53].

### 3.5. The Relationship between Sfp1 and Cas5 in Cell Wall Stress Response

Different regulatory patterns between two transcription factors have been reported in controlling various biological processes by modulation of target gene expression [54]. For example, one transcription factor may repress the activity of the other that normally activates gene expression. Alternatively, two transcription factors may coordinately or independently regulate the expression of a set of genes [54].

**Figure 5:**
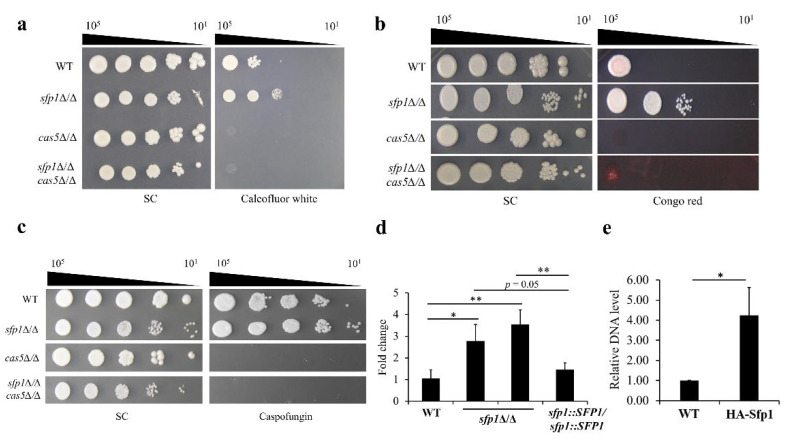
Sfp1 regulates cell wall stress response through Cas5. Cell susceptibility to (**a**) calcofluor white; (**b**) congo red; and (**c**) caspofungin was determined by spot assay. The cells were ten-fold serially diluted and spotted onto SC agar plates with or without calcofluor white (600 μg/mL), congo red (100 μg/mL) and caspofungin (1 μg/mL). The plates were incubated at 30 °C for 5 d. Representative images of three independent experiments with similar results are shown; (**d**) The expression of *CAS5* gene was detected using real-time qPCR. The *PMA1* transcripts were used as an internal control for the RNA input. Results are displayed as the mean ± SD for three independent experiments. The relative fold changes were displayed as the expression levels of an individual strain normalized to the WT strain (as 1). *, *p* < 0.05, **, *p* < 0.01; (**e**) The ChIP assay for binding of Sfp1 to the *CAS5* promoter. DNA fragments from the WT and HA-*SFP1* strains were amplified by real-time qPCR. Results are displayed as the mean ± SD for three independent experiments. *, *p* < 0.05.

Based on the findings of this work and other studies, Sfp1 and Cas5 are both involved in *C. albicans* response to cell wall stress. Interestingly, the *cas5*Δ/Δ mutant shows hypersensitivity to cell wall-disrupting agents, including congo red and caspofungin [16]. Nevertheless, unlike Cas5, deletion of *SFP1* leads to cell resistance to congo red, and calcofluor white (Figure 1a,b and Figure 2). According to these opposite phenotypes, determining whether a regulatory relationship exists between Sfp1 and Cas5 is therefore of interest. In order to address this question, the genetic relationship between Sfp1 and Cas5 in cell wall response was first established. For this purpose, the *CAS5* gene was deleted in the WT (i.e., *cas5*Δ/Δ) and in the *sfp1*-deletion mutant (i.e., *sfp1*Δ/Δ *cas5*Δ/Δ), separately. Susceptibility of the WT, *sfp1*Δ/Δ, *cas5*Δ/Δ and *sfp1*Δ/Δ *cas5*Δ/Δ mutants to cell wall-disrupting agents was examined using the spot assay. As shown in Figure 5a–c, the *sfp1*Δ/Δ mutant was resistant to all the cell wall-disrupting agents tested, whereas the *cas5*Δ/Δ mutants exhibited a hypersensitive phenotype. Interestingly, like the *cas5*Δ/Δ mutant, the *sfp1*Δ/Δ *cas5*Δ/Δ double deletion mutants were also hypersensitive to all the drugs tested. Additionally, the caspofungin MICs were also determined. The MIC50 for the *cas5*Δ/Δ and *sfp1*Δ/Δ *cas5*Δ/Δ mutant was 0.031 and 0.0625 µg/mL, respectively. Moreover, the caspofungin MIC90 for the *cas5*Δ/Δ and *sfp1*Δ/Δ *cas5*Δ/Δ mutant was 0.031 and 0.125 µg/mL, respectively. These results indicated that both *cas5*Δ/Δ and *sfp1*Δ/Δ *cas5*Δ/Δ mutants required fewer amounts of caspofungin to inhibit 50% and 90% of cells compared to the WT, *sfp1*Δ/Δ, and *SFP1*-reintegration strains (as shown in Table 1). Together, the epistasis relationship of Sfp1 and Cas5 suggested that one may repress the function of the other in response to cell wall stress. Otherwise, Sfp1 and Cas5 may function independently, but not coordinately.

### 3.6. SFP1 Controls Expression of CAS5 through Direct Binding of Sfp1 to the CAS5 Promoter

To further reveal the regulatory relationship between Sfp1 and Cas5, expression of *CAS5* was monitored using real-time qPCR. Intriguingly, expression of *CAS5* was significantly upregulated in the *sfp1*Δ/Δ mutants compared to the WT and *SFP1*-reintegration strains (Figure 5d). Moreover, to determine whether Sfp1 can directly regulate *CAS5* expression, ChIP analysis was performed and the resultant DNA carrying the *CAS5* promoter was quantified using real-time qPCR. The data showed that the DNA carrying the *CAS5* promoter was significantly enriched by HA-tagged Sfp1 compared to the WT strain carrying untagged Sfp1 (Figure 5e). Collectively, our results suggested that Sfp1 normally negatively regulates *CAS5* expression through its direct binding to the *CAS5* promoter element.

## 4. Discussion

The cell wall of *C*. *albicans* is a dynamic structure, playing a vital role to provide cell shape and rigidity, and participating in different biological processes. Moreover, the cell wall is the first point of *C*. *albicans* to interact with the host [5,55]. In response to environmental stresses, cells remodel their walls to maintain the structural and functional integrity of the cell wall.

*C. albicans* Sfp1 is a transcription factor involved in carbon source-stress adaptation, ribosome biogenesis, cell adhesion, biofilm formation, and oxidative stress response [24,25,26,56]. Moreover, Sfp1 was shown to play a role in cell wall and ER stress responses induced by LL-37 [35]. However, the intrinsic effect of Sfp1 on CWI has not yet been investigated. In this study, as demonstrated by spot assay, zymolyase digestion analysis, and CSH measurement, the *sfp1*Δ/Δ mutant exhibited alterations in cell wall-associated properties (Figure 1a–d). Moreover, the *sfp1*Δ/Δ mutant was more resistant to caspofungin (Figure 2). Finally, modulation in cell wall polysaccharide composition, cell wall structure, and cell wall-related gene expression was also revealed in the *sfp1*Δ/Δ mutant (Figure 3 and Figure 4). These results provide evidence that changes in cell wall coincided with deletion of *SFP1*, suggesting a link between Sfp1 and regulation of CWI maintenance.

Cas5 is a transcriptional regulator and deletion of *CAS5* renders hypersensitivity to cell wall-disrupting agents and caspofungin [16]. Moreover, Cas5 is able to suppress hyphal morphogenesis and is required for *C. albicans* virulence [57,58]. Interestingly, Cas5 is also involved in cell adhesion and biofilm formation [59,60]. To elucidate how Cas5 regulates CWI, several studies have focused on cellular response to cell wall stress, particularly that induced by caspofungin. Cas5 is activated by the phosphatase Glc7 and can cooperatively function with the transcriptional regulators Swi4 and Swi6 to control gene expression [17]. Moreover, Cas5 and the transcription factor Efg1 are shown to bind to some caspofungin-responsive gene promoters and orchestrate activation of these target gene expression [61]. Together, these studies indicate that Cas5 is a key regulator in *C. albicans* CWI and stress response. 

Since the opposite phenotype between the *sfp1*Δ/Δ and *cas5*Δ/Δ mutant in their susceptibility to cell wall disrupting agents and caspofungin [16] (Figure 1a,b and Figure 2), determining the relationship between Sfp1 and Cas5 is therefore of interest. Genetic interaction analysis showed that the *sfp1*Δ/Δ *cas5*Δ/Δ double deletion mutant exhibited hypersensitivity to calcofluor white, congo red and caspofungin, similar to that of the *cas5*Δ/Δ mutant but in contrast to that of the WT and *sfp1*Δ/Δ strains (Figure 5a–c). Moreover, real-time qPCR and ChIP assay further demonstrated that the level of *CAS5* transcript is upregulated in the *sfp1*Δ/Δ mutant and Sfp1 binds to the *CAS5* promoter (Figure 5d,e). Besides, whole-genome DNA microarray analysis has previously identified genes that are differentially expressed between the *sfp1*Δ/Δ and wild type strain [26]. Notably, revisiting the dataset of DNA microarray (the GEO accession number GSE127184), many of these differentially expressed genes are cell wall-related. For example, expression of *XOG1*, *PGA6*, *PGA38*, *FGR41*, *ALS3*, *SIM1*, *ORF19.1258*, *RBE1*, *PIR1*, *SCW11*, *ORF19.675,* and *YWP1* are upregulated in the *sfp1*Δ/Δ mutant (Table S3 in the Supplementary Materials). Intriguingly, in contrast to the expression pattern in the *sfp1*Δ/Δ mutant, this subset of genes is downregulated in the *cas5*Δ/Δ mutant [17]. Because Cas5 is known to activate many cell wall biosynthesis and remodeling genes, our results suggest that Sfp1 regulates cell wall-related target genes and exerts its effect on CWI partly through its negative control of the *CAS5* gene expression. However, whether Sfp1 is related to Cas5 in other functions such as cell adhesion, biofilm formation and cell cycle dynamics still needs to be determined.

Apart from Cas5, Sfp1, Swi4, Swi6 and Efg1, other transcription factors, such as Rlm1 and Sko1 are also involved in *C. albicans* caspofungin-induced cell wall stress response [62,63,64]. Interestingly, Rlm1 was reported to indirectly regulate Sko1 in response to cell wall stress [62,64]. Moreover, using ChIP-sequence analysis, Sko1 was shown to bind to the *EFG1* upstream intergenic region under cell wall stress, suggesting Sko1 may directly regulate *EFG1* [64]. Although the relationship between Sfp1 and Efg1 in regulation of CWI is not yet established, Sfp1 suppresses biofilm formation through negative regulation of Efg1 [24]. In combination with the findings that Cas5 acts in concert with Swi4, Swi6, and Efg1, regulatory network in maintenance of CWI is complex and requires positive and negative interactions among different transcription factors. Therefore, to further understand the function of Sfp1 relevant to CWI, the mutual interaction of Sfp1 with Swi4, Swi6, Efg1, Rlm1, and Sko1 remains to be elucidated.

Importantly, other than transcription regulators, signaling cascades are also essential in *C*. *albicans* response and adaptation to environmental changes [65,66,67]. For example, cell response to cell wall stress is mediated through different signaling pathways, including the protein kinase C (PKC) and mitogen-activated protein kinase (MAPK) Mkc1 cascade [65,66]. Interestingly, deletion of *CAS5* leads to the activation of the Mkc1 CWI pathway even in the absence of cell wall stress [17]. Moreover, the high osmolarity glycerol (HOG) and Cek1 MAPK pathways also play a role in cell wall reconstruction [68,69]. Of note, the MAPK Hog1 of the HOG pathway is associated with Sfp1 in *C*. *albicans* growth and cell size regulation [56]. As demonstrated in *S*. *cerevisiae*, the unfolded protein response (UPR) signaling from the ER is induced by the CWI pathway and is required for CWI maintenance [70]. Interestingly, our recent study showed that Sfp1 coordinates the cellular responses to ER and cell wall stress induced by LL-37 [23]. Besides, deletion of *SFP1* brings about an increased phosphorylation level of Mkc1 in cell response to LL-37 [23]. Finally, the calcium-calcineurin pathway crosstalks with the MAPK Mkc1 pathway in response to caspofungin [71]. However, the connection between Sfp1, other cell wall stress-responsive transcription factors, and the calcium-calcineurin pathway have not been studied. Further analysis of the association between Sfp1 and different signaling pathways is needed.

In conclusion, the cell wall is essential in *C*. *albicans*, providing cell shape, involving different biological processes, and directly interacting with the host. Maintenance of CWI in response to environmental stress is thus critical for cell survival and pathogenicity of *C*. *albicans*. Here, we not only elucidate a previously unknown function of Sfp1 in CWI but also show a negative control of the *CAS5* gene expression by Sfp1. Cas5 is a transcription factor lacking an ortholog in *S*. *cerevisiae* and humans and is required for antifungal resistance and *C*. *albicans* virulence. To our knowledge, this is the first report to identify a negative regulator of *CAS5* transcription. Moreover, this study also highlights a regulatory network of CWI involving a complex interplay between signaling and transcription factors.

## Figures and Tables

**Table 1:** Minimal inhibitory concentrations (MICs) of caspofungin.

Strains	MIC50 (μg/mL)	MIC90 (μg/mL)
SC5314	0.125	0.25
*sfp1*Δ/Δ	0.25	>8
*sfp1*Δ*::SFP1/sfp1*Δ*::SFP1*	0.125	0.25

## Data Availability

The data presented in this study are available within the article and the Supplementary Materials.

## Supplementary Materials

The following supporting information can be downloaded at: https://www.mdpi.com/article/10.3390/jof8111196/s1, Table S1: *C. albicans* strains used in this study; Table S2: Primers used in this study; Table S3: Differential regulation of a subset of genes by Sfp1 and Cas5. Figure S1: Images of three independent experiments of spot assay shown in Figure 1a,b, Figure 2, Figure 5a–c. Reference [72] is cited in the supplementary materials.

## References

[1] Wisplinghoff H., Bischoff T., Tallent S.M., Seifert H., Wenzel R.P., Edmond M.B. (2004). Nosocomial bloodstream infections in US hospitals: Analysis of 24,179 cases from a prospective nationwide surveillance study. Clin. Infect. Dis..

[2] Brown G.D., Denning D.W., Gow N.A., Levitz S.M., Netea M.G., White T.C. (2012). Hidden killers: Human fungal infections. Sci. Transl. Med..

[3] Perlin D.S., Rautemaa-Richardson R., Alastruey-Izquierdo A. (2017). The global problem of antifungal resistance: Prevalence, mechanisms, and management. Lancet Infect. Dis..

[4] Gow N.A., Hube B. (2012). Importance of the *Candida albicans* cell wall during commensalism and infection. Curr. Opin. Microbiol..

[5] Garcia-Rubio R., de Oliveira H.C., Rivera J., Trevijano-Contador N. (2020). The fungal cell wall: *Candida*, *Cryptococcus*, and *Aspergillus* species. Front. Microbiol..

[6] Lenardon M.D., Sood P., Dorfmueller H.C., Brown A.J., Gow N.A. (2020). Scalar nanostructure of the *Candida albicans* cell wall; a molecular, cellular and ultrastructural analysis and interpretation. Cell Surf..

[7] Gow N.A.R., Latge J.P., Munro C.A. (2017). The Fungal Cell Wall: Structure, Biosynthesis, and Function. Microbiol. Spectr..

[8] Dranginis A.M., Rauceo J.M., Coronado J.E., Lipke P.N. (2007). A biochemical guide to yeast adhesins: Glycoproteins for social and antisocial occasions. Microbiol. Mol. Biol. Rev..

[9] Hoyer L.L., Cota E. (2016). *Candida albicans* agglutinin-like sequence (Als) family vignettes: A review of Als protein structure and function. Front. Microbiol..

[10] Gow N.A., Van De Veerdonk F.L., Brown A.J., Netea M.G. (2012). *Candida albicans* morphogenesis and host defence: Discriminating invasion from colonization. Nat. Rev. Microbiol..

[11] de Groot P.W., Bader O., de Boer A.D., Weig M., Chauhan N. (2013). Adhesins in human fungal pathogens: Glue with plenty of stick. Eukaryot. Cell.

[12] Bain J.M., Louw J., Lewis L.E., Okai B., Walls C.A., Ballou E.R., Walker L.A., Reid D., Munro C.A., Brown A.J. (2014). *Candida albicans* hypha formation and mannan masking of β-glucan inhibit macrophage phagosome maturation. mBio.

[13] Szymański M., Chmielewska S., Czyżewska U., Malinowska M., Tylicki A. (2022). Echinocandins–structure, mechanism of action and use in antifungal therapy. J. Enzym. Inhib. Med. Chem..

[14] Ene I.V., Walker L.A., Schiavone M., Lee K.K., Martin-Yken H., Dague E., Gow N.A., Munro C.A., Brown A.J. (2015). Cell wall remodeling enzymes modulate fungal cell wall elasticity and osmotic stress resistance. mBio.

[15] Walker L.A., Munro C.A. (2020). Caspofungin induced cell wall changes of *Candida* species influences macrophage interactions. Front. Cell Infect. Microbiol..

[16] Bruno V.M., Kalachikov S., Subaran R., Nobile C.J., Kyratsous C., Mitchell A.P. (2006). Control of the *C. albicans* cell wall damage response by transcriptional regulator Cas5. PLoS Pathog..

[17] Xie J.L., Qin L., Miao Z., Grys B.T., Diaz J.D., Ting K., Krieger J.R., Tong J., Tan K., Leach M.D. (2017). The *Candida albicans* transcription factor Cas5 couples stress responses, drug resistance and cell cycle regulation. Nat. Commun..

[18] Heredia M.Y., Gunasekaran D., Ikeh M.A.C., Nobile C.J., Rauceo J.M. (2020). Transcriptional regulation of the caspofungin-induced cell wall damage response in *Candida albicans*. Curr. Genet..

[19] den Hertog A.L., van Marle J., van Veen H.A., Van’t Hof W., Bolscher J.G., Veerman E.C., Nieuw Amerongen A.V. (2005). Candidacidal effects of two antimicrobial peptides: Histatin 5 causes small membrane defects, but LL-37 causes massive disruption on the cell membrane. Biochem. J..

[20] Tsai P.W., Yang C.Y., Chang H.T., Lan C.Y. (2011). Human antimicrobial peptide LL-37 inhibits adhesion of *Candida albicans* by interacting with yeast cell-wall carbohydrates. PLoS ONE.

[21] Tsai P.W., Yang C.Y., Chang H.T., Lan C.Y. (2011). Characterizing the role of cell-wall beta-1,3-exoglucanase Xog1p in *Candida albicans* adhesion by the human antimicrobial peptide LL-37. PLoS ONE.

[22] Tsai P.W., Cheng Y.L., Hsieh W.P., Lan C.Y. (2014). Responses of *Candida albicans* to the human antimicrobial peptide LL-37. J. Microbiol..

[23] Hsu C.M., Liao Y.L., Chang C.K., Lan C.Y. (2021). *Candida albicans* Sfp1 is involved in the cell wall and endoplasmic reticulum stress responses induced by human antimicrobial peptide LL-37. Int. J. Mol. Sci..

[24] Chen H.F., Lan C.Y. (2015). Role of *SFP1* in the regulation of *Candida albicans* biofilm formation. PLoS ONE.

[25] Kastora S.L., Herrero-de-Dios C., Avelar G.M., Munro C.A., Brown A.J. (2017). Sfp1 and Rtg3 reciprocally modulate carbon source-conditional stress adaptation in the pathogenic yeast *Candida albicans*. Mol. Microbiol..

[26] Lee S.Y., Chen H.F., Yeh Y.C., Xue Y.P., Lan C.Y. (2019). The transcription factor Sfp1 regulates the oxidative stress response in *Candida albicans*. Microorganisms.

[27] Reuss O., Vik A., Kolter R., Morschhauser J. (2004). The SAT1 flipper, an optimized tool for gene disruption in *Candida albicans*. Gene.

[28] Yang Y.L., Wang C.W., Leaw S.N., Chang T.P., Wang I.C., Chen C.G., Fan J.C., Tseng K.Y., Huang S.H., Chen C.Y. (2012). R432 is a key residue for the multiple functions of Ndt80p in *Candida albicans*. Cell Mol. Life Sci..

[29] Sandini S., Stringaro A., Arancia S., Colone M., Mondello F., Murtas S., Girolamo A., Mastrangelo N., De Bernardis F. (2011). The MP65 gene is required for cell wall integrity, adherence to epithelial cells and biofilm formation in *Candida albicans*. BMC Microbiol..

[30] de Souza R.D., Mores A.U., Cavalca L., Rosa R.T., Samaranayake L.P., Rosa E.A. (2009). Cell surface hydrophobicity of *Candida albicans* isolated from elder patients undergoing denture-related candidosis. Gerodontology.

[31] Arendrup M.C., Cuenca-Estrella M., Lass-Flörl C., Hope W., EUCAST-AFST (2012). EUCAST technical note on the EUCAST definitive document EDef 7.2: Method for the determination of broth dilution minimum inhibitory concentrations of antifungal agents for yeasts EDef 7.2 (EUCAST-AFST). Clin. Microbiol. Infect..

[32] Desnos-Ollivier M., Bretagne S., Boullié A., Gautier C., Dromer F., Lortholary O., French Mycoses Study Group (2019). Isavuconazole MIC distribution of 29 yeast species responsible for invasive infections (2015–2017). Clin. Microbiol. Infect..

[33] Francois J.M. (2006). A simple method for quantitative determination of polysaccharides in fungal cell walls. Nat. Protoc..

[34] Plaine A., Walker L., Da Costa G., Mora-Montes H.M., McKinnon A., Gow N.A., Gaillardin C., Munro C.A., Richard M.L. (2008). Functional analysis of *Candida albicans* GPI-anchored proteins: Roles in cell wall integrity and caspofungin sensitivity. Fungal Genet. Biol..

[35] Hsu P.C., Yang C.Y., Lan C.Y. (2011). *Candida albicans* Hap43 is a repressor induced under low-iron conditions and is essential for iron-responsive transcriptional regulation and virulence. Eukaryot Cell..

[36] Livak K.J., Schmittgen T.D. (2001). Analysis of relative gene expression data using real-time quantitative PCR and the 2(−Delta Delta C(T)) Method. Methods.

[37] Bates S., Hughes H.B., Munro C.A., Thomas W.P., MacCallum D.M., Bertram G., Atrih A., Ferguson M.A., Brown A.J., Odds F.C. (2006). Outer chain N-glycans are required for cell wall integrity and virulence of *Candida albicans*. J. Biol. Chem..

[38] Hazen K.C. (1989). Participation of yeast cell surface hydrophobicity in adherence of *Candida albicans* to human epithelial cells. Infect. Immun..

[39] Masuoka J., Hazen K.C. (2004). Cell wall mannan and cell surface hydrophobicity in *Candida albicans* serotype A and B strains. Infect. Immun..

[40] Sumiyoshi M., Miyazaki T., Makau J.N., Mizuta S., Tanaka Y., Ishikawa T., Makimura K., Hirayama T., Takazono T., Saijo T. (2020). Novel and potent antimicrobial effects of caspofungin on drug-resistant *Candida* and bacteria. Sci. Rep..

[41] Nather K., Munro C.A. (2008). Generating cell surface diversity in *Candida albicans* and other fungal pathogens. FEMS Microbiol. Lett..

[42] Dichtl K., Samantaray S., Wagener J. (2016). Cell wall integrity signalling in human pathogenic fungi. Cell Microbiol..

[43] Reyna-Beltrán E., Isaac Bazán Méndez C., Iranzo M., Mormeneo S., Pedro Luna-Arias J. (2019). The cell wall of *Candida albicans*: A proteomics view. Candida albicans.

[44] Mio T., Adachi-Shimizu M., Tachibana Y., Tabuchi H., Inoue S.B., Yabe T., Yamada-Okabe T., Arisawa M., Watanabe T., Yamada-Okabe H. (1997). Cloning of the *Candida albicans* homolog of *Saccharomyces cerevisiae GSC1/FKS1* and its involvement in beta-1, 3-glucan synthesis. J. Bacteriol..

[45] Douglas C.M., D’ippolito J.A., Shei G.J., Meinz M., Onishi J., Marrinan J.A., Li W., Abruzzo G.K., Flattery A., Bartizal K. (1997). Identification of the *FKS1* gene of *Candida albicans* as the essential target of 1, 3-beta-D-glucan synthase inhibitors. Antimicrob. Agents Chemother..

[46] del Mar González M., Díez-Orejas R., Molero G., Pla J., Nombela C., Sánchez-PéArez M. (1997). Phenotypic characterization of a *Candida albicans* strain deficient in its major exoglucanase. Microbiology.

[47] Childers D.S., Avelar G.M., Bain J.M., Pradhan A., Larcombe D.E., Netea M.G., Erwig L.P., Gow N.A., Brown A.J. (2020). Epitope shaving promotes fungal immune evasion. mBio.

[48] Lenardon M.D., Munro C.A., Gow N.A. (2010). Chitin synthesis and fungal pathogenesis. Curr. Opin. Microbiol..

[49] Mio T., Yabe T., Sudoh M., Satoh Y., Nakajima T., Arisawa M., Yamada-Okabe H. (1996). Role of three chitin synthase genes in the growth of *Candida albicans*. J. Bacteriol..

[50] Munro C.A., Winter K., Buchan A., Henry K., Becker J.M., Brown A.J., Bulawa C.E., Gow N.A. (2001). Chs1 of *Candida albicans* is an essential chitin synthase required for synthesis of the septum and for cell integrity. Mol. Microbiol..

[51] Lenardon M.D., Whitton R.K., Munro C.A., Marshall D., Gow N.A. (2007). Individual chitin synthase enzymes synthesize microfibrils of differing structure at specific locations in the *Candida albicans* cell wall. Mol. Microbiol..

[52] Walker L.A., Munro C.A., De Bruijn I., Lenardon M.D., McKinnon A., Gow N.A. (2008). Stimulation of chitin synthesis rescues *Candida albicans* from echinocandins. PLoS Pathog..

[53] Walker L.A., Gow N.A., Munro C.A. (2013). Elevated chitin content reduces the susceptibility of *Candida* species to caspofungin. Antimicrob. Agents Chemother..

[54] Zheng J., Benschop J.J., Shales M., Kemmeren P., Greenblatt J., Cagney G., Holstege F., Li H., Krogan N.J. (2010). Epistatic relationships reveal the functional organization of yeast transcription factors. Mol. Syst. Biol..

[55] Ruiz-Herrera J., Victoria Elorza M., Valentín E., Sentandreu R. (2006). Molecular organization of the cell wall of *Candida albicans* and its relation to pathogenicity. FEMS Yeast Res..

[56] Sellam A., Chaillot J., Mallick J., Tebbji F., Richard Albert J., Cook M.A., Tyers M. (2019). The p38/HOG stress-activated protein kinase network couples growth to division in *Candida albicans*. PLoS Genet..

[57] Kim J.M., Moon H.Y., Lee D.W., Kang H.A., Kim J.Y. (2021). The transcription factor Cas5 suppresses hyphal morphogenesis during yeast-form growth in *Candida albicans*. J. Microbiol..

[58] Chamilos G., Nobile C.J., Bruno V.M., Lewis R.E., Mitchell A.P., Kontoyiannis D.P. (2009). *Candida albicans* Cas5, a regulator of cell wall integrity, is required for virulence in murine and toll mutant fly models. J. Infect. Dis..

[59] Finkel J.S., Xu W., Huang D., Hill E.M., Desai J.V., Woolford C.A., Nett J.E., Taff H., Norice C.T., Andes D.R. (2012). Portrait of *Candida albicans* adherence regulators. PLoS Pathog..

[60] Ponde N.O., Lortal L., Ramage G., Naglik J.R., Richardson J.P. (2021). *Candida albicans* biofilms and polymicrobial interactions. Crit. Rev. Microbiol..

[61] Xiong K., Su C., Sun Q., Lu Y. (2021). Efg1 and Cas5 orchestrate cell wall damage response to caspofungin in *Candida albicans*. Antimicrob Agents Chemother.

[62] Delgado-Silva Y., Vaz C., Carvalho-Pereira J., Carneiro C., Nogueira E., Correia A., Carreto L., Silva S., Faustino A., Pais C. (2014). Participation of *Candida albicans* transcription factor *RLM1* in cell wall biogenesis and virulence. PLoS ONE.

[63] Rauceo J.M., Blankenship J.R., Fanning S., Hamaker J.J., Deneault J.S., Smith F.J., Nantel A., Mitchell A.P. (2008). Regulation of the *Candida albicans* cell wall damage response by transcription factor Sko1 and PAS kinase Psk1. Mol. Biol. Cell.

[64] Heredia M.Y., Ikeh M.A., Gunasekaran D., Conrad K.A., Filimonava S., Marotta D.H., Nobile C.J., Rauceo J.M. (2020). An expanded cell wall damage signaling network is comprised of the transcription factors Rlm1 and Sko1 in *Candida albicans*. PLoS Genet..

[65] Ibe C., Munro C.A. (2021). Fungal cell wall proteins and signaling pathways form a cytoprotective network to combat stresses. J. Fungi.

[66] Sanz A.B., García R., Rodríguez-Peña J.M., Arroyo J. (2017). The CWI pathway: Regulation of the transcriptional adaptive response to cell wall stress in yeast. J. Fungi.

[67] Sanz A.B., García R., Pavón-Vergés M., Rodríguez-Peña J.M., Arroyo J. (2022). Control of gene expression via the yeast CWI pathway. Int. J. Mol. Sci..

[68] Eisman B., Alonso-Monge R., Roman E., Arana D., Nombela C., Pla J. (2006). The Cek1 and Hog1 mitogen-activated protein kinases play complementary roles in cell wall biogenesis and chlamydospore formation in the fungal pathogen *Candida albicans*. Eukaryot. Cell.

[69] Monge R.A., Roman E., Nombela C.P.L.A., Pla J. (2006). The MAP kinase signal transduction network in *Candida albicans*. Microbiology.

[70] Scrimale T., Didone L., de Mesy Bentley K.L., Krysan D.J. (2009). The unfolded protein response is induced by the cell wall integrity mitogen-activated protein kinase signaling cascade and is required for cell wall integrity in *Saccharomyces cerevisiae*. Mol. Biol. Cell.

[71] da Silva Dantas A., Nogueira F., Lee K.K., Walker L.A., Edmondson M., Brand A.C., Lenardon M.D., Gow N.A. (2021). Crosstalk between the calcineurin and cell wall integrity pathways prevents chitin overexpression in *Candida albicans*. J. Cell Sci..

[72] Gillum A.M., Tsay E.Y., Kirsch D.R. (1984). Isolation of the Candida albicans gene for orotidine-5’-phosphate decarboxylase by complementation of *S. cerevisiae ura3* and *E. coli pyrF* mutations. Mol. Gen. Genet. MGG.

