# Supplementary material for: The Role of Sfp1 in Candida albicans Cell Wall Maintenance
Source: J Fungi (Basel). 2022 Nov 13;8(11):1196. doi: 10.3390/jof8111196 (PMC9692975; doi:10.3390/jof8111196)
Supplement: Supplementary file 1 [file jof-08-01196-s001.zip › jof-1985325-supplementary.pdf]

Supplementary Materials

Table S1. *C. albicans* strains used in this study.

Strain Name	Genotype	Source
SC5314	Wild type	[1]
*sfp1*Δ/Δ 1-3	*sfp1*Δ::*FRT*/*sfp1*Δ::*FRT*	[2]
*sfp1*Δ/Δ 6-17	*sfp1*Δ::*FRT*/*sfp1*Δ::*FRT*	[2]
SFP1-reintegration	*sfp1*Δ::*SFP1-FRT*/ *sfp1*Δ::*SFP1-FRT*	[2]
*cas5*Δ/Δ	*cas5*Δ::*FRT*/*cas5*Δ::*FRT*	This study
*sfp1*Δ/Δ *cas5*Δ/Δ	*sfp1*Δ::*FRT*/*sfp1*Δ::*FRT*<br>*cas5*Δ::*FRT*/ *cas5*Δ::*FRT*	This study
HA-*SFP1*	*sfp1*Δ::*FRT*/*sfp1*Δ::*FRT*<br>*ENO1*/*ENO1*::HA-*SFP1*	This study

Table S2. Primers used in this study.

Primer Name	Sequence (5′ to 3′) [a]
**For real-time qPCR**	
qPMA1-F	TTGCTTATGATAATGCTCCATACGA
qPMA1-R	TACCCCACAATCTTGGCAAGT
qFKS1-F	TGATACTGGTAATCATAGACCAAAAA
qFKS1-R	AACTCTGAATGGATTTGTAGAATAAGG
qXOG1-F	GGGTTGTTATGCTAAAAAGGA
qXOG1-R	AATCTGTCAAAGCAGCAGACCAT
qCHS1-F	TTGGAACCGG TGGAACATCT
qCHS1-R	AGACCATAGGTGGACAACAATGAA
qCHS3-F	TGTTTGTTCCTAAAGCTGCTTGTAA
qCHS3-R	AACGACGACGTTGCGATAATAA
qCHS8-F	AAACGAATGCTGATGGATCCA
qCHS8-R	CCAGCGACAATCCATAAAATGA
qSFP1-F	GCACGTCACCTCTACGTTATGG
qSFP1-R	CGATGACCGGGCACTTG
CAS5-qF1	TGAAAACTCCTCTTCGAGGTCAT
CAS5-qR2	GCAGCAGTAACAGCAGCATTG
**For strain construction**	
CAS5UR-F-KpnI	GTGAGGTACCCATCCACCCCCTACAATCAA
CAS5UR-R-XhoI	GACCCTCGAGGGTGGGATGAAAAGCAAAGT
CAS5DR-F-SacII	GCTTCCGCGGTGAATGTCAAATGCCAAACTG
CAS5DR-F-SacI	GCAGGAGCTCTCATCATGCGAGTTATGGTTT
SphI-HA-SFP1-F	AGCATGCtacgacgtaccagattacgcttacgacgtaccagattacgcttacgac-<br>gtaccagattacgctTTTAATACCAAGATATTTGAAAATACAGG
SphI-HA-SFP1-R[a]	AGCATGCTTAATGAGTGGTATGCCCAC
**For ChIP**	
CAS5 promoter qF	CAATATAAGCGGGTTATCTAGAATTAGAC
CAS5 promoter qR	ATCCACTAGTTTAGGAAATACCTAATAAAC
ADE2 promoter qF	AATGAGCTGAATCCACGACAAA
ADE2 promoter qR	CACGTGACCCAAGAGTTTTAATGT

[a] The restriction sites are underlined and lowercase letters denote the HA tag sequence.

Table S3. Differential regulation of a subset of genes by Sfp1 and Cas5.

Orf19 ID	Gene Name	*sfp1*Δ/Δ v.s WT (Relative Fold Change)[a,b]	*cas5*Δ/Δ v.s WT (Relative Fold Change)[c]
**Glucan synthase & Glucanase**			
orf19.2990	*XOG1*	26.25	-2.8
**GPI-anchored proteins**			
orf19.4765	*PGA6*	1.71	-2.1
orf19.2758	*PGA38*	1.62	-3
orf19.4910	*FGR41*	1.55	-2.78
**Adhesin**			
orf19.1816	*ALS3*	1.51	-1.73
orf19.5032	*SIM1*	2.57	-1.5
orf19.1258	*C4_05730W_A*	2.69	-5.16
**Others**			
orf19.4670	*CAS5*	1.96	-27.78
orf19.7218	*RBE1*	2.97	-2.73
orf19.220	*PIR1*	1.53	-2.2
orf19.3893	*SCW11*	2.11	-3.1
orf19.3618	*YWP1*	1.96	-2.92
orf19.675	*C1_11270W_A*	2.45	-2.09

[a] Lee, S. Y., Chen, H. F., Yeh, Y. C., Xue, Y. P., & Lan, C. Y. (2019). The transcription factor Sfp1 regulates the oxidative stress response in *Candida albicans*. Microorganisms, 7(5), 131. [b] The GEO accession number GSE127184. [c] Xie, J. L., Qin, L., Miao, Z., Grys, B. T., Diaz, J. D. L. C., Ting, K., ... & Cowen, L. E. (2017). The *Candida albicans* transcription factor Cas5 couples stress responses, drug resistance and cell cycle regulation. Nature Communications, 8(1), 1-18.

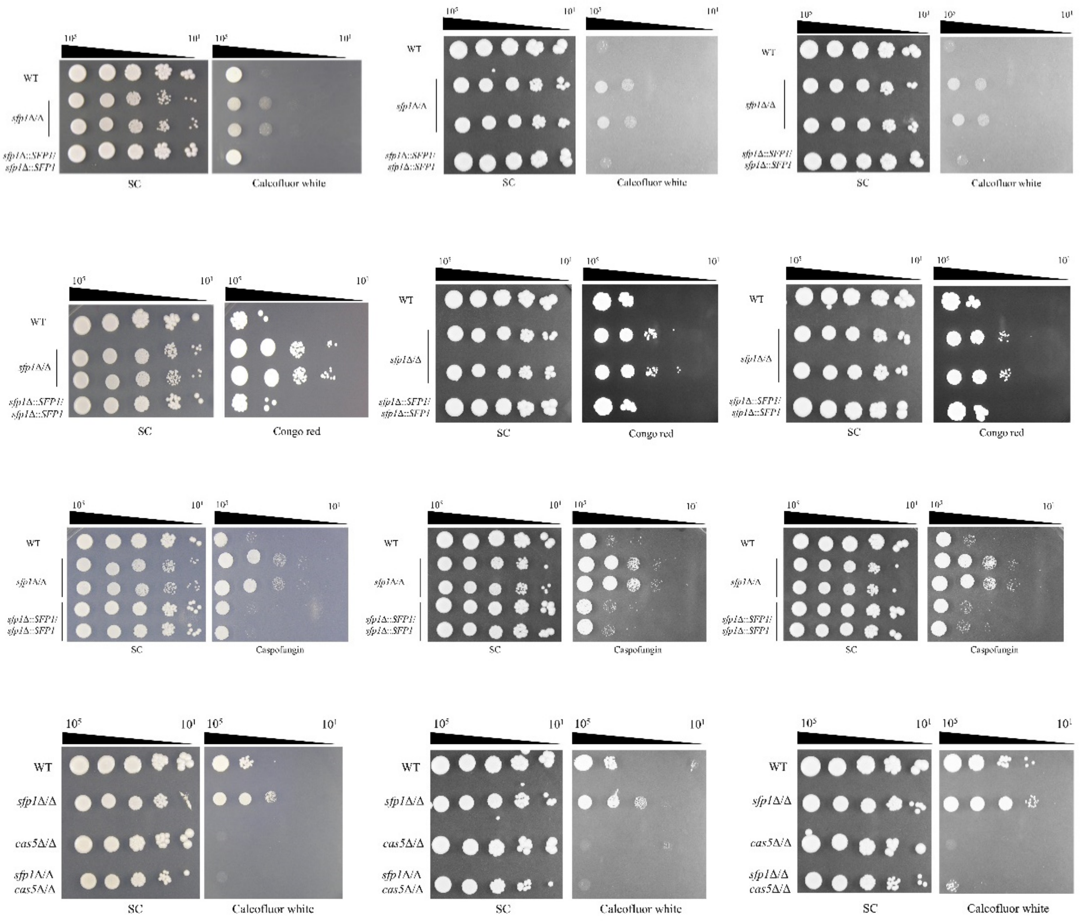

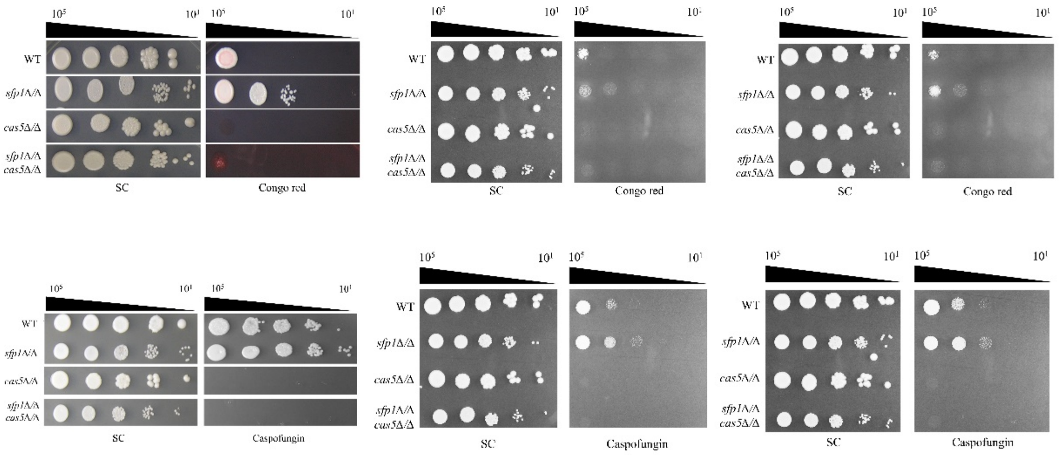

Figure S1. Images of three independent experiments of spot assay shown in Figures 1a,b, 2 and 5a–c.

References

1. Gillum, A.M.; Tsay, E.Y.; Kirsch, D.R. Isolation of the *Candida albicans* gene for orotidine-5′-phosphate decarboxylase by complementation of *S. cerevisiae ura3* and *E. coli pyrF* mutations. *Mol. Gen. Genet.* **1984**, *198*, 179–182.
2. Chen, H.F.; Lan, C.Y. Role of *SFP1* in the regulation of *Candida albicans* biofilm formation. *PLoS ONE* **2015**, *10*, e0129903.
